# Population-specific variations in *KCNH2* predispose patients to delayed ventricular repolarization upon dihydroartemisinin-piperaquine therapy

**DOI:** 10.1128/aac.01390-23

**Published:** 2024-03-28

**Authors:** Mahamadou D. Camara, Yitian Zhou, Antoine Dara, Mamadou M. Tékété, Taís Nóbrega de Sousa, Sékou Sissoko, Laurent Dembélé, Nouhoun Ouologuem, Amadou Hamidou Togo, Mohamed L. Alhousseini, Bakary Fofana, Issaka Sagara, Abdoulaye A. Djimde, Pedro J. Gil, Volker M. Lauschke

**Affiliations:** 1 Department of Physiology and Pharmacology, Karolinska Institutet, Stockholm, Sweden; 2 Department of Epidemiology of Parasitic Diseases, Malaria Research and Training Center, Faculty of Pharmacy, University of Science, Techniques and Technologies, Bamako, Mali; 3 Department of Microbiology and Tumour Cell Biology, Karolinska Institutet, Stockholm, Sweden; 4 Molecular Biology and Malaria Immunology Research Group, Instituto René Rachou, Fundação Oswaldo Cruz (FIOCRUZ), Belo Horizonte, Brazil; 5 Global Health and Tropical Medicine, Institute of Hygiene and Tropical Medicine, Nova University of Lisbon, Lisbon, Portugal; 6 Dr. Margarete Fischer-Bosch Institute of Clinical Pharmacology, Stuttgart, Germany; 7 University of Tübingen, Tübingen, Germany; The Children's Hospital of Philadelphia, Philadelphia, Pennsylvania, USA

**Keywords:** malaria, cardiotoxicity, long QT, QT prolongation, hERG, Kv11.1

## Abstract

Dihydroartemisinin-piperaquine is efficacious for the treatment of uncomplicated malaria and its use is increasing globally. Despite the positive results in fighting malaria, inhibition of the Kv11.1 channel (hERG; encoded by the *KCNH2* gene) by piperaquine has raised concerns about cardiac safety. Whether genetic factors could modulate the risk of piperaquine-mediated QT prolongations remained unclear. Here, we first profiled the genetic landscape of *KCNH2* variability using data from 141,614 individuals. Overall, we found 1,007 exonic variants distributed over the entire gene body, 555 of which were missense. By optimizing the gene-specific parametrization of 16 partly orthogonal computational algorithms, we developed a *KCNH2*-specific ensemble classifier that identified a total of 116 putatively deleterious missense variations. To evaluate the clinical relevance of *KCNH2* variability, we then sequenced 293 Malian patients with uncomplicated malaria and identified 13 variations within the voltage sensing and pore domains of Kv11.1 that directly interact with channel blockers. Cross-referencing of genetic and electrocardiographic data before and after piperaquine exposure revealed that carriers of two common variants, rs1805121 and rs41314375, experienced significantly higher QT prolongations (ΔQTc of 41.8 ms and 61 ms, respectively, vs 14.4 ms in controls) with more than 50% of carriers having increases in QTc >30 ms. Furthermore, we identified three carriers of rare population-specific variations who experienced clinically relevant delayed ventricular repolarization. Combined, our results map population-scale genetic variability of *KCNH2* and identify genetic biomarkers for piperaquine-induced QT prolongation that could help to flag at-risk patients and optimize efficacy and adherence to antimalarial therapy.

## INTRODUCTION

Dihydroartemisinin-piperaquine (DHA-PQ) combination therapy, one of the frontline treatments for uncomplicated malaria, is becoming increasingly used throughout Africa (1). Besides prophylaxis in children and adults, DHA-PQ is also suggested for intermittent preventive treatment in pregnancy either alone (2) or in combination with azithromycin (3). Importantly, piperaquine is structurally similar to chloroquine, which is a well-known cause of significant electrophysiological aberrations and cardiovascular toxicity (4). While meta-analyses have not confirmed an increased risk of lethal cardiac events (5), piperaquine was significantly associated with delayed ventricular repolarization in different cohorts undergoing DHA-PQ chemoprevention (6–8).

Like chloroquine, piperaquine has been shown to block the Kv11.1 ion channel (commonly referred to as hERG and encoded by the *KCNH2* gene), which is critical for cardiac repolarization (9). Kv11.1 is a voltage-gated potassium channel that is comprised of a homotetramer of four ⍺-subunits (10). Each ⍺-subunit contains an N-terminal Per-Arnt-Sim (PAS) domain, six transmembrane domains (S1–S6), and a C-terminal cyclic nucleotide-binding domain (cNBD). The transmembrane domains are functionally differentiated into the voltage sensor domain (VSD) from S1 to S4 and the pore domain formed by S5 and S6. Upon activation, the cytoplasmic gate opens slowly in response to membrane depolarization resulting in potassium fluxes through the pore (11). Subsequent repolarization results in rapid channel closure and inactivation, which terminates fluxes at depolarized membrane potentials and allows to generate the resurgent current during repolarization. The primary voltage-sensing structure in the S4 domain is activated after membrane depolarization and is responsible for the slow opening process followed by fast inactivation (12). The blockade of the protein in this state leads to a prolongation of the QT interval (13), which in turn constitutes an important risk factor for potentially lethal ventricular tachyarrhythmia, such as Torsade de pointes (14).

Notably, Kv11.1 activity is influenced by various demographic and biochemical parameters, such as sex, age, and electrolyte levels (15, 16). Furthermore, genetic variability has been shown to be associated with the sensitivity to drug-induced QT prolongations (17). Examples include p.R784W, which reduces the rapid rectifier potassium current (I_kr_) through the channel upon amiodarone exposure (18), p.R1047L that predisposes to dofetilide-induced Torsade de pointes (19), as well as multiple variants associated with long QT syndrome in the general population at baseline (20, 21). Importantly, little information is available about the overall landscape of genetic variability of *KCNH2* at the population scale, particularly in African populations, which remain underrepresented in pharmacogenetic research (22).

Here, we assessed the genetic variability of *KCNH2* using publicly available sequencing data from 141,614 unrelated individuals across 12 human populations. In total, we identified 1,007 distinct variations distributed across all channel domains. Using an array of 16 computational variant effect predictors, we estimate that approximately 1 in 130 individuals carries at least one *KCNH2* variant with functional effects that might predispose to QT prolongation. To assess the functional importance of genetic Kv11.1 variability and contribute toward profiling of the genetic diversity in Africa, we sequenced *KCNH2* of 293 patients with uncomplicated malaria from Kollé and Bougoula Hameau in Mali who received artemisinin-based combination therapies. We identified a total of 13 variants in the pharmacologically important S4 helix and pore domain of which five were ultra-rare with global frequencies <0.0001 and an additional four were novel. Importantly, we found that, while piperaquine levels were a poor predictor of cardiotoxicity upon DHA-PQ therapy, a common *KCNH2* haplotype as well as three rare variants were associated with clinically significant QT prolongations. Taken together, these results map the landscape of *KCNH2* variability in different ethnogeographic groups and identify population-specific variations that significantly associate with adverse events upon DHA-PQ treatment.

## MATERIALS AND METHODS

### Population-scale genetic data

Genetic variability data for *KCNH2* (Ensembl gene ID ENSG00000055118) from a total of 141,614 unrelated individuals were extracted from gnomAD (23). The data set encompassed information from 8,128 Africans and African Americans, 17,296 admixed Americans, 5,040 Ashkenazi Jews, 9,197 East Asians, 10,824 Finns, 56,885 non-Finnish Europeans, and 15,308 South Asians. The gnomAD data set was aggregated from different projects and reprocessed using uniform pipelines to increase consistency. Cohorts recruited for pediatric disease are not included. The overall population is balanced between males and females. Variant identification was based on reference sequence NM_000238.4. Variant carrier frequency was calculated by aggregating variant frequencies using the Hardy-Weinberg equation. Variants with minor allele frequencies (MAFs) ≥1% were considered as common while variants with MAFs <1% were defined as rare.

### Variant effect predictions

The calibration data set of 96 deleterious and 37 neutral *KCNH2* missense variants with known effects were extracted from ClinVar. Specifically, we considered variants annotated with “Pathogenic” or “Likely pathogenic” as deleterious and variants annotated with “Benign” or “Likely benign” as neutral. The functional effect of missense variants of unknown significance was predicted using 16 computational algorithms developed for the identification of pathogenic variations: M-CAP (24), MetaLR and MetaSVM (25), MVP (26), MutationTaster2 (27), CADD (28), Eigen (29), MetaRNN (30), ClinPred (31), PolyPhen-2 (32), LRT (33), SIFT (34), PROVEAN (35), MutPred (36), MutationAssessor (37), and FitCons (38). These tools base their assessments on a variety of partly orthogonal features, including evolutionary conservation, structural information, allele frequency, and functional genomic data. Parameter optimization based on receiver operating characteristic (ROC) curves was conducted in R (version 4.3.0). The results of all optimized algorithms were integrated into an ensemble score and a variant was assumed to be deleterious if >50% of the *KCNH2*-optimized algorithms predicted deleteriousness. In addition, we considered frameshift, stop-gain, start-lost, and canonical splice donor and acceptor variants as putatively deleterious.

### Study cohorts from Mali

We took advantage of the previous WANECAM trial (West African Network for Clinical Trials of Anti-Malarial Drugs, registry number: PACTR201105000286876), a randomized, multinational, multicenter, open-label, longitudinal, controlled phase 3b/4 clinical trial, to analyze associations between cardiotoxicity and genetic variations (39). Within WANECAM, the safety and efficacy of multiple artemisinin-based combination therapies were evaluated, including DHA-PQ. In short, after inclusion, each enrolled patient was followed for a total of 2 years and received the same treatment at each new episode according to the study protocol. In the DHA-PQ arm, patients had an active follow-up period of 63 days for each episode after inclusion. Before commencing treatment, clinical and parasitological parameters were measured. A 12-lead electrocardiogram (ECG) was recorded before treatment (day 0) and at day 2, 4–6 hours after the last dose, to assess DHA-PQ cardiotoxicity. QT prolongation was measured using a method independent from heart rate changes and from the method of QT correction, which has been successfully used for the analysis of QT prolongation in artemisinin combination therapies (40). At day 7, veinous blood was collected to measure piperaquine concentrations. Dried blood spots were collected for genetic analyses. In total, our genetic analyses included data from 95 and 198 patients, respectively, from Kollé and Bougoula Hameau, Mali. The demographic and clinical parameters of the study population are provided in Table 1. For cardiotoxicity associations, we considered all patients with available drug exposure data, resulting in a subset of 69 patients treated with DHA-PQ (Fig. S1).

**TABLE 1:** Baseline demographic and clinical features of the analyzed trial cohort^*a*^

Parameters	Kollé	Bougoula Hameau
Number of patients	95	198
Female	44 (46.3 %)	100 (50.5 %)
Age in years (SD)	8 (3.5)	8 (3.6)
<5	20 (21.1 %)	42 (21.2 %)
≥5 to <15	74 (77.9 %)	154 (77.8)
≥15	1 (1.1 %)	2 (1 %)
Body weight in kg (SD)	22.44 (7.6)	22 (9)
<20	41 (43.2 %)	95 (48 %)
≥20	54 (56.8 %)	103 (52 %)
Fever present	95 (100 %)	190 (96 %)
Body temperature in °C (SD)	37.3 (1)	37.9 (1.1)
*Plasmodium falciparum* asexual forms	90 (94.7 %)	191 (96 %)
Median number of parasites per microliter (IQR)	22,360 (3,760–60,620)	16,470 (925–39,285)
*Plasmodium ovale* asexual forms	2 (2.1 %)	0
Median number of parasites per microliter (IQR)	1,380 (1,020–1,740)	0
*Plasmodium malariae* asexual forms	3 (3.2 %)	7 (4 %)
Median number of parasites per microliter (IQR)	1,960 (1,130–2,100)	880 (158–3,790)
Patients with gametocytes		
*Plasmodium falciparum*	2	11 (6 %)
*Plasmodium malariae*	0	0
*Plasmodium ovale*	0	0

^*a*^ IQR, interquartile range; SD, standard deviation.

### DNA extraction and sequencing

We used dried blood spots produced from peripheral blood on filter paper to extract genomic DNA using column-based extraction kits (QIAGEN). From the extracted DNA, we amplified two fragments of 492 bp and 321 bp encoding the S4 helix and pore domain as reported previously (41). The amplified fragments were sequenced at the Karolinska Institutet Genomic Core Facility (KIgene, Solna, Sweden) on an ABI 3730 PRISM DNA Analyzer (Applied Biosystems). We used an inhouse bioinformatic pipeline developed by the Malaria Research and Training Center (Mali) to generate the consensus sequences based on EMBOSS and MAFFT. The result was analyzed with BioEdit (v7.2.5) and Unipro UGENE (v48.0).

### Estimation of linkage disequilibrium

PLINK 2.0 (42) was used to calculate linkage disequilibria at the *KCNH2* locus (±100 kb of the transcription start site). Linkages were calculated based on data from the 1000 Genomes Project phase 3 and variants with *R*^2^ >0.2 were considered to be linked.

### Statistical analysis

Statistical tests were conducted in RStudio (v2023.06.2+561) and Prism (v8.4.3). Normality was tested using Shapiro Wilk tests. Changes in QTc between genotype groups were compared using Kruskal-Wallis tests. Association between drug level and QTc variation was evaluated using linear regression analysis. *P*-values <0.05 were considered significant.

## RESULTS

### Population-scale variability of KCNH2

**Fig 1:**
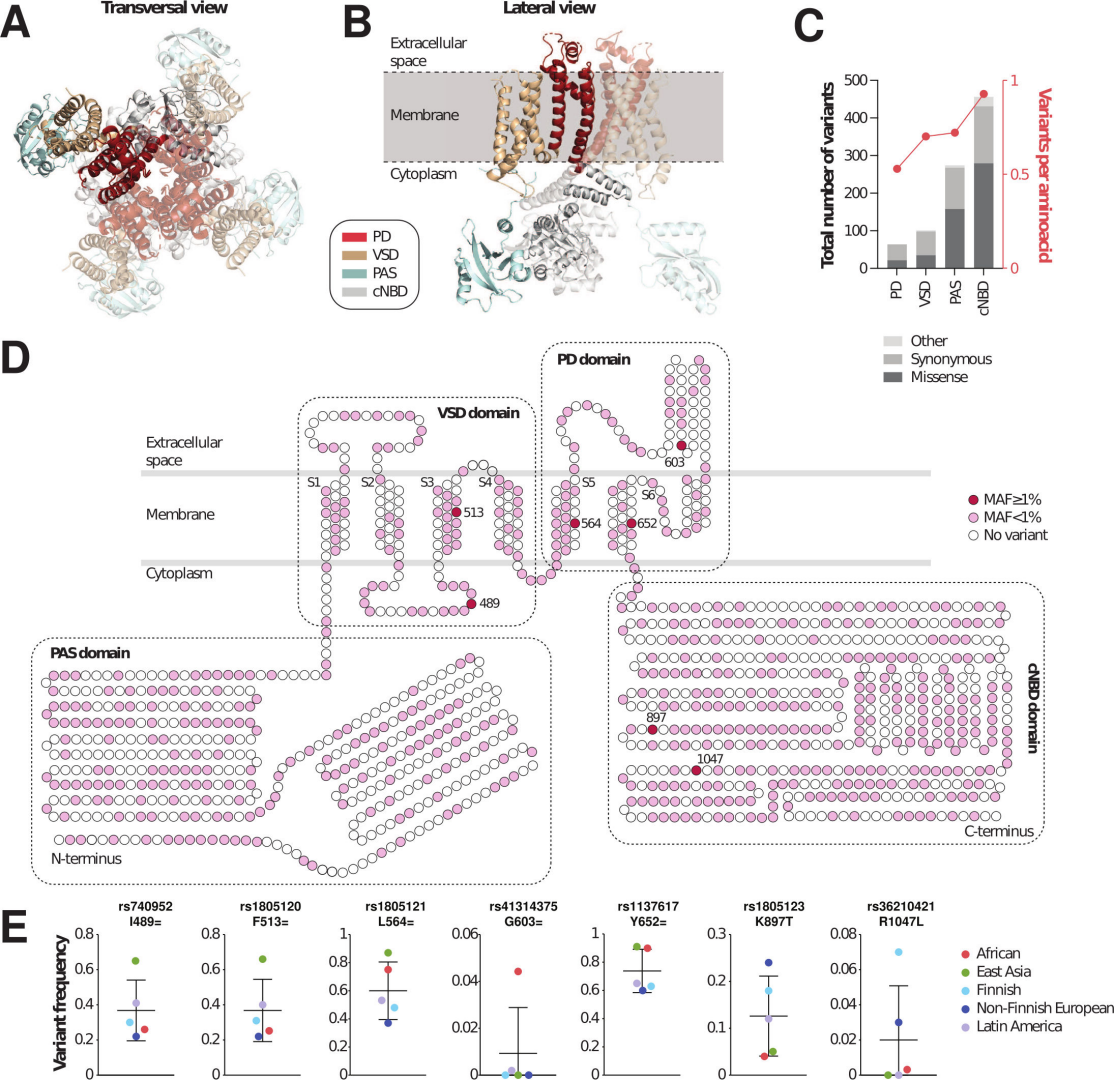
The landscape of genetic variability of *KCNH2* (hERG). The 3D structure of Kv11.1 is shown in transversal (**A**) and lateral views (**B**). One of the four identical subunits is shown as opaque and the other three as transparent. The PD, VSD, PAS, and cNBD are shown in red, brown, turquoise, and gray, respectively. (C) The total number of variants (left y-axis; column plot) and the number of variants per amino acid (right y-axis; dot plot) are shown for each domain. (D) Schematic representation of the distribution of variants across protein domains. All common variations in *KCNH2* are shown in dark red, all rare variants in magenta. (E) The frequencies of all common *KCNH2* variations are shown across five major ethnogeographic groups. Mean and standard deviations across the indicated five populations (Africans, East Asians, Finnish, non-Finnish Europeans, and Latin Americans) are shown.

The Kv11.1 channel is formed by four identical ⍺-subunits, each 1,159 amino acids long comprising a transmembrane VSD and pore domain (PD), as well as a cytoplasmic PAS and cNBD (Fig. 1A and B). Overall, the encoding gene *KCNH2* is highly polymorphic. Analysis of genomic information from 141,614 individuals revealed a total of 1,007 exonic variants of which 20 are frameshifts, 14 are in-frame indels, 555 are missense, 400 are synonymous, and 18 are stop-gain, start-lost, or splice variants. Most variants are found in the cNBD and PAS domains, whereas the functionally most important PD and VSD harbor around fourfold fewer total variants (Fig. 1C). Variation rates were between 0.5 (PD) and 1 (cNBD) variant per amino acid. Notably, while some amino acids were impacted by multiple different variations, others were not affected by variants.

Of all identified variations, only seven were common in at least one major population (Fig. 1D). Two common variants each were located in the VSD and cNBD, while three mapped to the PD. Notably, the common variants exhibited high variability in prevalence across the different populations (Fig. 1E). Overall, most common were rs1137617 and rs1805121 with global frequencies of 66.7% and 48.4%, respectively. The variants rs740952 and rs1805120 were in complete linkage and formed a haplotype that was most common in East Asian populations (MAF = 65%). Rs41314375, rs1805123, and rs1137617 were population-specific and differed in frequency more than fivefold between ethnogeographic groups. Based on these data, we conclude that the genetic variability of *KCNH2* is extensive with a plethora of rare and population-specific variations.

### Computational variant effect predictions suggest that functional variability in KCNH2 is widespread

**Fig 2:**
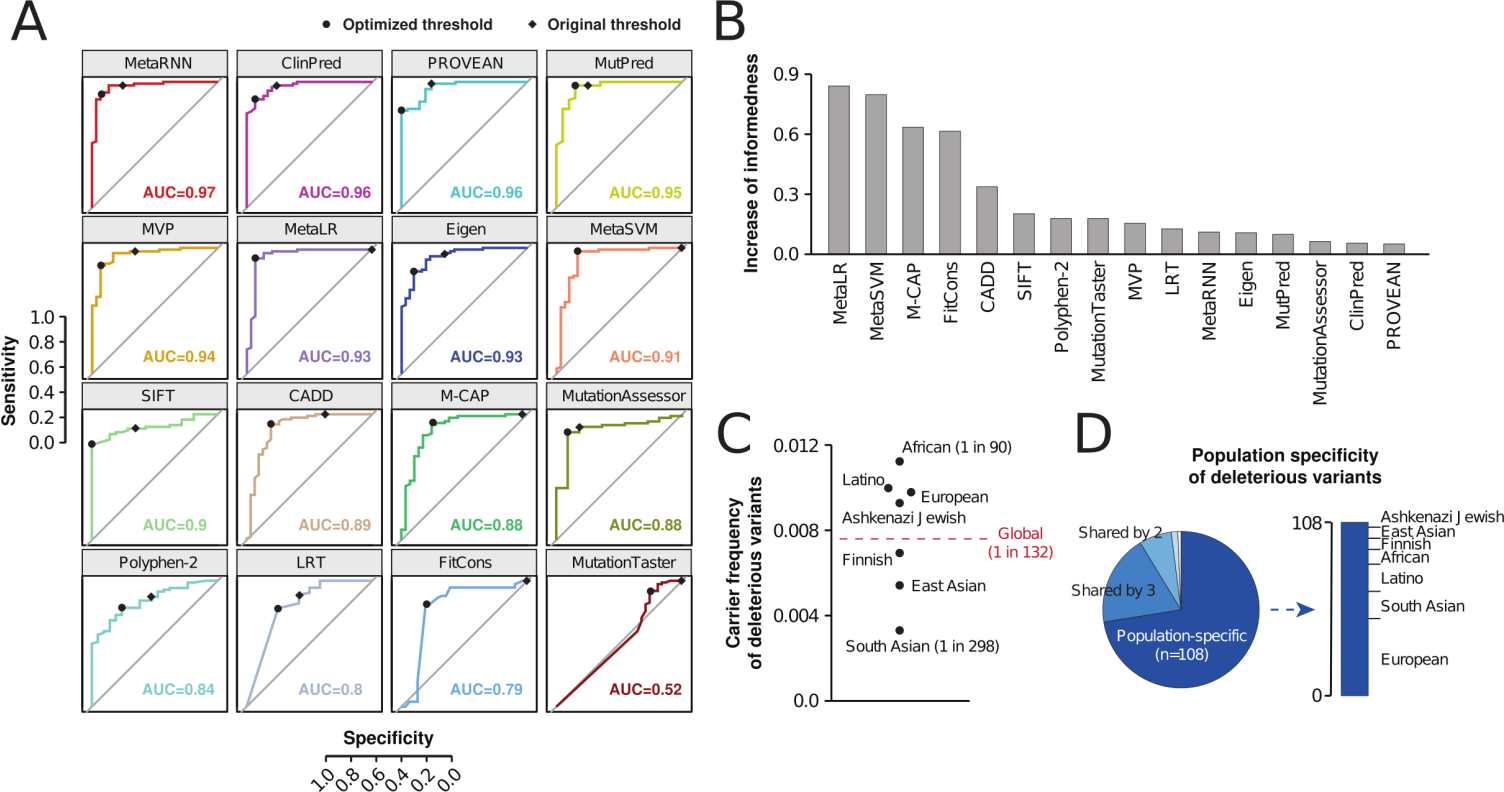
The *KCNH2* gene harbors a multitude of rare and population-specific variations that are predicted to have deleterious effects on channel function. (A) ROC curves of sensitivity (ordinate) vs 1-specificity (abscissa) are shown for 16 computational variant effect predictors. Areas under the ROC curves (AUC) are indicated for each algorithm. Dots indicate the performance when using the original parameters, while diamonds indicate performance after informedness optimization. (B) The increase in informedness upon parameter optimization is shown for each algorithm. (C) The carrier frequency of deleterious *KCNH2* variants, obtained by aggregating the population-specific frequencies of all putatively deleterious variants, is shown for different populations. Dashed line indicates the global average based on genomic data from 141,614 individuals. (D) Pie chart showing that the majority of deleterious *KCNH2* variations (72%) were only identified in a single population. Due to the population imbalance of available genomic data, the largest number of population-specific variations is presently identified in Europeans (stacked column plot).

Next, we leveraged 16 commonly used variant effect predictors to parse the functional impacts of the identified genetic variability. We evaluated algorithm performance on a set of 96 deleterious and 37 neutral *KCNH2* variants from ClinVar (Table 2). MetaRNN and ClinPred achieved the overall best performance (74% informedness), whereas MetaLR, MetaSVM, MutationTaster, and FitCons (0% informedness) were not able to discriminate between deleterious and benign variations using predefined parameters (Fig. 2A). We then optimized the parametrization of each algorithm to maximize informedness. To this end, we used the Youden index (J), which was originally devised to rate the quality of diagnostic tests (43) but has also been used for the optimization of pharmacogenomic prediction algorithms (44). Youden’s J is calculated on the basis of ROC curves for all potential threshold scores *x* and is defined as

**TABLE 2:** Performance of computational variant effect predictors on *KCNH2* variants with known functional effects

Algorithm	Original	Spec	Sens	J	Optimized	Spec	Sens	J	AUC
MetaRNN	≥0.5	0.77	0.97	0.74	≥0.885	0.93	0.92	0.85	0.97
ClinPred	≥0.5	0.77	0.97	0.74	≥0.982	0.93	0.86	0.79	0.96
PROVEAN	<−2.282	0.76	0.96	0.72	<−3.87	1	0.77	0.77	0.96
MutPred	>0.5	0.75	0.97	0.72	>0.587	0.85	0.97	0.82	0.95
MVP	>0.75	0.67	0.97	0.64	>0.942	0.93	0.86	0.79	0.94
MetaLR	≥0.5	0	1	0	≥0.913	0.93	0.92	0.85	0.93
Eigen	≥0	0.68	0.93	0.61	≥0.3675	0.9	0.81	0.71	0.93
MetaSVM	≥0	0	1	0	≥0.8535	0.83	0.97	0.8	0.91
SIFT	≤0.05	0.67	0.89	0.55	≤0.0025	1	0.76	0.76	0.9
CADD	>15	0.39	1	0.39	>22.9	0.81	0.92	0.73	0.89
M-CAP	≥0.025	0.03	1	0.03	≥0.6895	0.76	0.92	0.68	0.88
MutationAssessor	>1.9	0.82	0.89	0.7	>2.0225	0.91	0.86	0.77	0.88
PolyPhen-2	>0.452	0.52	0.84	0.37	>0.674	0.76	0.79	0.55	0.84
LRT	<0.001	0.58	0.82	0.41	<0.0005	0.75	0.78	0.53	0.8
FitCons	>0.4	0	1	0	>0.6905	0.81	0.81	0.62	0.79
MutationTaster	>0.5	0	1	0	>0.974	0.26	0.92	0.18	0.52

^*a*^ AUC, area under the receiver operating characteristic curve; Original, parameters defined in the respective original publications (see Materials and Methods section for references); Optimized, KCNH2-specific parameters after informedness optimization; Spec, specificity; Sens, sensitivity; J, Youden’s J.

*J* = max_x_ {sensitivity(x) + specificity(x) − 1}

The optimized parameter is then selected so that the sum of sensitivity and speciﬁcity is maximal, thus balancing sensitivity and speciﬁcity and avoiding impacts of the unequal distribution of neutral and functionally deleterious variants in the available training data (45). Optimization improved the performance of all algorithms (Fig. 2B). For MetaLR, MetaSVM, M-CAP, and FitCons, informedness was increased by >60%, whereas gains for already well-performing algorithms, such as PROVEAN and ClinPred, were expectedly smaller (<10%). Lastly, prediction scores of the optimized algorithms were integrated into a binary consensus classification that was then used for the classifications of *KCNH2* variants of unknown functional significance.

Based on this gene-optimized ensemble score, 116 of the identified 555 missense variants were predicted to have deleterious effects. When aggregating their frequencies, 1 in 132 individuals can be expected to harbor at least one deleterious genetic variation in *KCNH2* (Fig. 2C). The highest number of deleterious variations was found in Africans (1 in 90 individuals), whereas deleterious missense variants in *KCNH2* were rarer in South Asian (1 in 298) and East Asian (1 in 183) populations. Thus, while individually rare, the overall burden of deleterious missense variants in *KCNH2* is relatively high, which could put patients at increased risk of developing QT prolongation upon exposure to drugs that interact with Kv11.1. Importantly, the vast majority of these variants (72%) were population-specific (Fig. 2D). While missense variants can be computationally assessed, the functional impact of non-coding variations cannot be reliably predicted using current tools and effects of such variations are likely to further increase the number of individuals with predisposing variations. Given the large extent of population-specific variability, these results emphasize the need to consider ethnogeographic variability when studying *KCNH2* variability.

### Common genetic variants are significantly associated with QT prolongation upon piperaquine exposure

To evaluate the clinical relevance of these implications, we focused on an African patient population undergoing DHA-PQ therapy for uncomplicated malaria. To this end, we first evaluated the genetic variability of *KCNH2* in 293 patients recruited within the WANECAM trial across two different sites in Mali (Kollé and Bougoula Hameau), approximately 350 km apart. We focused our analyses specifically on the VSD and PD domains, as these have been shown to directly interact with Kv11.1 blockers, specifically with residues 624–628 (46). In total, we identified 13 variants of which seven were missense variants and the other six were synonymous (Table 3). Among the identified variants, three were common with frequencies of the ancestral allele between 4.3% and 16.4% in the Malian population and 10 were rare (MAF <1%). Furthermore, four novel variations were identified. Variant frequencies were overall similar in the two study sites, the only exception being rs1805121, for which the A allele had a frequency of 19.6% in Kollé and 14.8% in Bougoula Hameau (Table S1). Notably, our *KCNH2* variant effect predictor classified five of the seven missense variants as deleterious.

**TABLE 3:** *KCNH2* genotype and allele distribution in the Malian and other populations

SNPs ref^*b*^	HGVS consequence	Protein changes	Freq Mali (%)	Freq Africa (%)	Freq global (%)	*KCNH2* predictor
rs1805121	c.1692A > G	p.Leu564=	83.56	75.43	48.42	Uncertain
rs41314375	c.1809C > T	p.Gly603=	4.84	4.42	4.3	Uncertain
rs1137617	c.1956T > C	p.Tyr652=	95.70	89.55	66.71	Uncertain
rs1335240034	c.1653C > G	p.Phe551Leu	0.17	0	0.0032	Neutral
rs199473517	c.1689G > T	p.Trp563Cys	0.17	NA^*a*^	0.324	Deleterious
rs1460801598	c.1701C > G	p.Ile567Met	0.17	0	0.0004	Deleterious
rs121912508	c.1744C > T	p.Arg582Cys	0.17	0	0.1	Deleterious
rs150275982	c.1581G > A	p.Ala527=	0.17	0.302	0.032	Uncertain
rs2116964399	c.1596G > C	p.Leu532=	0.17	NA	NA	Uncertain
NM_000238.4:c.1637G > A		p.Gly546Ser	0.17	NA	NA	Deleterious
NM_000238.4:c.1604T > A		p.Val535Ala	0.17	NA	NA	Deleterious
NM_000238.4:c.1989T > A		p.Ile663Asn	0.17	NA	NA	Uncertain
NM_000238.4:c.2118C > T		p.Ser706=	0.17	NA	NA	Uncertain

^*a*^ NA, no information available;

^*b*^ SNP = single nucleotide polymorphism. HGVS = Human Genome Variation Society.

**Fig 3:**
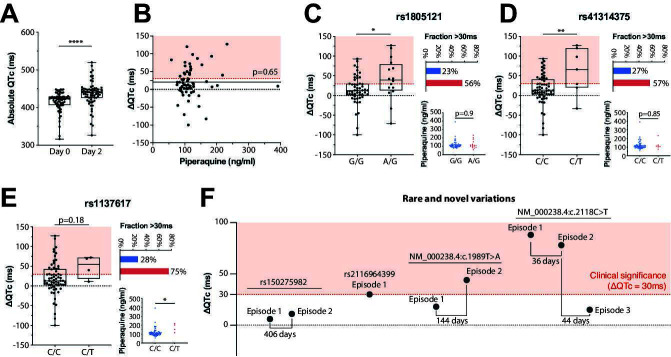
Association of identified populations with QT prolongation. (A) Absolute QTc intervals in milliseconds (ms) measured before (day 0) and after 2 days of dihydroartesunate piperaquine combination therapy. **** indicates *P* < 0.0001 using a Kruskal-Wallis test. (B) The variation in QTc from day 0 to day 2 (ΔQTc) was not associated with measured serum piperaquine levels. The black dashed line indicates no change in QTc intervals (ΔQTc = 0), the red dashed line indicates an increase in QTc above the clinically significant threshold of 30 ms, and the black solid line constitutes the regression line (*P* = 0.65 for deviation of slope from zero). (C–E) The variation in QTc from day 0 to day 2 (ΔQTc) is shown for carriers of the common variations rs1805121 (**C**), rs41314375 (**D**), and rs1137617 (**E**). The upper inlets show the fraction of variant carriers (red) and controls (blue) who experience QT prolongations >30 ms. The lower inlets show serum piperaquine concentrations in carriers (red) and controls (blue). * and ** indicate *P* < 0.05 and *P* < 0.01 using a Kruskal-Wallis test. (F) QTc interval variations are shown for carriers of rare and novel *KCNH2* variations for successive episodes. Time difference between episodes is indicated.

All patients included in the study were examined by ECG, both before commencing DHA-PQ therapy and after 2 days of treatment. At baseline, the median QTc was 423 ms (95% CI: 415–426 ms; Fig. 3A). Patients with QTc >450 ms were excluded. At day 2, we observed a statistically significant increase of the QTc interval (*P* < 0.00001; median QTc = 440 ms; 95% CI: 433–444 ms), indicating an overall drug-induced QT prolongation (ΔQTc) at the cohort level (median ΔQTc = 15 ms; range: −100 to +127 ms). Overall, 21 out of 69 patients (30.4%) had clinically significant QT prolongations, defined as >30 ms. The extent of QT prolongation was not correlated with piperaquine levels (*R*^2^ = 0.005; *P* = 0.65), suggesting that non-pharmacokinetic factors might contribute to the observed delays in ventricular repolarization (Fig. 3B).

We next evaluated whether common genetic factors could explain at least part of the observed effects on cardiac electrophysiology. In total, piperaquine levels were available for 53 homozygous and 16 heterozygous carriers of rs1805121 (Fig. 3C). The median ΔQTc upon DHA-PQ treatment in patients with the G/G diplotype was 13 ms (95% CI: 4–23 ms), whereas ΔQTc in A/G individuals was significantly higher (41.8 ms; 95% CI: 11–83 ms; *P* = 0.019). The fraction of patients with ΔQTc >30 ms, was 56% in rs1805121 carriers compared to 23% in controls. As for the overall cohort, no association with piperaquine concentrations was identified (*P* = 0.9). Variant rs41314375 is closely linked to rs1805121 with all seven carriers harboring both variants in one haplotype. Furthermore, both variants were in strong linkage (*R*^2^ >0.2) with a total of 59 other variations at the *KCNH2* locus (Table S3), complicating the attribution of the observed effects to individual variations. In rs41314375 carriers, the QTc variation was significantly higher than in controls (61 ms; 95% CI: 6.6–115.4 ms vs 16.2 ms; 95% CI: 6.7–25.6 ms; *P* = 0.006) with 57% compared to 27% of patients experiencing ΔQTc >30 ms (Fig. 3D). In addition, a trend toward increased QT prolongation was observed for rs1137617 (53.5 ms; 95% CI: 2.5–93.5 ms vs 13 ms; 95% CI: 8–27 ms; Fig. 3E). However, this trend was not statistically significant (*P* = 0.11), possibly due to the low number of identified variant carriers (*n* = 4). When we increased cohort size by also including individuals without available exposure information, we found similar but not statistically significant trends of increased QT prolongations in variant carriers with average increases in ΔQTc for rs1805121, rs41314375, and rs1137617 carriers of 5.2 ms, 19.6 ms, and 4.3 ms, respectively (Fig. S2; Table S2).

### QT prolongation in carriers of rare *KCNH2* variations

Four individuals with available electrophysiology data and piperaquine levels carried rare or novel *KCNH2* variants (Fig. 3F). The carrier of rs150275982 did not experience clinically significant QTc prolongations at either of the two episodes separated by 406 days (ΔQTc_episode1_ = 6 ms; ΔQTc_episode2_ = 11 ms). In contrast, one patient carrying both the rs2116964399 variant as well as the novel variant NM_000238.4:c.1604T > A showed a QT prolongation of 30 ms after 2 days of DHA-PQ therapy. The highest increases in QTc intervals were observed in two patients carrying the novel variants NM_000238.4:c.1989T > A and NM_000238.4:c.2118C > T. The patient heterozygous for NM_000238.4:c.1989T > A had a QTc variation during episode 1 of 18 ms, which increased to 44 ms during the second episode 144 days later (absolute QTc = 462 ms). The patient heterozygous for NM_000238.4:c.2118C > T featured very high QT prolongation of 88, 74, and 16 ms during three episodes. While statistical conclusions for these personal variations cannot be drawn from the data, these data provide case reports that some carriers of rare *KCNH2* variations experience substantial and clinically significant increases of QTc intervals upon piperaquine exposure.

## DISCUSSION

Cardiotoxicity constitutes a key concern in drug development. *In vitro* testing for Kv11.1 binding is mandated by regulatory authorities before a compound can enter clinical trials but, nevertheless, induction of cardiovascular adverse events remained the main reasons of safety failures in clinical trials and post-marketing surveillance (47, 48). Thus, optimization of drug candidates to minimize interaction with Kv11.1 constitutes an important aim in drug development; however, for some pharmacophores, Kv11.1 inhibition can hardly be avoided (49, 50). One class of such drugs are quinoline antimalarials, including chloroquine and piperaquine. The effect of quinolines on the prolongation of the QTc interval is well-established; yet, it is also clear that cardiac effects are highly variable between individuals. Understanding of the molecular underpinnings that predispose to quinoline cardiotoxicity and, ideally, the identification of biomarkers to predict individual risk thus constitute important goals of the safety pharmacology of this class of antimalarials. Here, we identified significant associations between piperaquine-induced changes in QT interval and two common synonymous variations in *KCNH2*, rs1805121 and rs41314375, and provide further case evidence for significant effects in three carriers of ultra-rare variants.

Genetic variants in *KCNH2* are a known cause of congenital long QT syndrome 2 (OMIM 613688), a rare autosomal dominant cardiac disease with prevalence around 1 in 6,000 live births (51). Furthermore, more frequent *KCNH2* variants have been shown to be associated with milder forms of QT prolongation (52). Importantly, most variations in *KCNH2* are rare and lack prior evidence regarding their pathogenicity. We thus established a gene-specific computational effect predictor to infer variant functionality by optimizing the parametrization of 16 state-of-the-art algorithms and integrating their assessments into a binary ensemble classifier. Similar approaches to design algorithms specific for variant predictions of a single gene had previously been successful for *TP53* (53), *DPYD* (54), and *CYP2D6* (55), where they exhibited improved performance compared to general purpose methods. Our ensemble score outperformed previous tools, suggesting that it might be suitable also for the prospective prediction of novel *KCNH2* variations with unknown functional consequences. Moreover, it could be interesting to assess score performance for variations within the 39 other human potassium channel genes to evaluate whether prediction accuracy can be generalized within this important gene family.

Experimental evidence indicates that *KCNH2* variants not only impact baseline electrophysiology but also increase the susceptibility to drug-induced arrhythmias (56). However, clinical evidence for such pharmacogenetic effects in patient cohorts exposed to Kv11.1 blockers is limited. Among rs1805121 and rs41314375 carriers, the fraction of individuals experiencing QT prolongations >30 ms was considerably higher, suggesting that these individuals are at increased risk for Torsade de pointes and subsequent sudden cardiac death. Rs1805121 and rs41314375 might thus be biomarker candidates for preemptive genetic testing in Mali.

Multiple previous studies have investigated associations between rs1805121 and QT interval either directly or indirectly by focusing on other variants in linkage with rs1805121. Strauss et al. identified the G allele of rs1805121 as a common genetic polymorphism contributing to long QTc at baseline in Europeans, whereas we, here, identified the A allele as the risk allele (57). However, the same study identified pronounced differences in the directionality of allele risk between Africans and Europeans, suggesting that effects can be population-specific. Zhang et al. did not identify associations of rs1805121 with nonfamilial cardiac arrhythmia in Chinese; however, ECG phenotypes were diverse and not focused on QT interval (58). Lastly, Pfeufer et al. found that the A allele of rs1805121 occurred in different haplotypes of which the one with the A allele of rs1805123, which was associated with longer QT interval, was the most common (59). Importantly however, none of these studies evaluated variant effects on drug-induced QT prolongation. These results thus incentivize replication studies of the identified associations and aim to stimulate investigations into possible associations between *KCNH2* variability and effects on drug-induced cardiac electrophysiology in other ethnogeographic groups. We deem such studies to be of major importance for public health considering that piperaquine is used in mass drug administration regimens, i.e., indiscriminate exposure of otherwise healthy subjects, as well as for nationwide prophylaxis programs, like intermittent preventive treatment in pregnancy (60) and malaria seasonal chemotherapy in children (61).

Synonymous variations have long been assumed to have minimal impacts on the functionality of the respective gene product. However, mounting evidence over the last decades has demonstrated that synonymous variations can alter transcription, splicing, as well as protein translation and folding (62–64). Specifically, for *KCNH2*, synonymous variations have been shown to alter trafficking and translation efficacy (65). Furthermore, synonymous *KCNH2* variations that exchange rare to common codons can cause misfolding resulting in non-functional Kv11.1 protein (66). While the functional consequences of the synonymous variations identified here have as of yet not been analyzed experimentally, rs1805121 indeed exchanges the rarest leucine codon CTA (frequency 6.9/1,000) to the most common codon CTG (frequency 40.3/1000)(67). It is thus plausible to speculate that the observed phenotypic effects associated with rs1805121 could potentially be mediated by increased misfolding of the resulting variant gene product. Given the high frequencies of the A allele of rs1805121 (16.5%), these results raise the possibility that this synonymous variation by itself does not have strong phenotypic consequences; however, upon challenge with a Kv11.1 blocker, the minor functional impairment would be amplified resulting in a measurable increase in adverse cardiac event risk. Notably, the *KCNH2*-specific ensemble score we presented here can in its current state only interpret missense variations. Thus, further extension that would also allow the evaluation of non-coding variations constitutes an important goal for future research.

An alternative explanation could be that the identified variants are only proxies for the functionally responsible variations. Both rs1805121 and rs41314375 are in complex linkage disequilibrium with other variations at the *KCNH2* locus, including intronic, as well as upstream and downstream variants with potential regulatory effects. As such, the available data do not allow to directly attribute the observed effects to the identified variations, and further functional studies are required to pinpoint the functionally responsible variations.

The presented work is, to the best of our knowledge, the first of its kind that describes the genetic variability of *KCNH2* in Mali. While we included two different study sites (Kollé and Bougoula Hameau) approximately 350 km apart with different autochtone ethnic groups, the results can only serve as a proxy for the Malian population. Further population-scale genetic data from a wider range of Malian ethnic groups would be required to increase generalizability. One of the key findings of our work was that two *KCNH2* variants were significantly associated with QT prolongation, whereas piperaquine exposure did not explain cardiotoxicity. This conclusion is based on an absence of correlation between ΔQTc and piperaquine concentration after 7 days of treatment. This time point was selected as it is commonly linked with malaria treatment efficacy; however, it cannot be excluded that earlier pharmacokinetic differences, e.g., differences in *c*_max_ or area under the exposure curve after day 2, might underlie at least part of the inter-individual variability in ΔQTc.

Combined, we developed an optimized gene-specific ensemble algorithm that accurately distinguishes deleterious and benign *KCNH2* variations. Application of this computational tool to population-scale genomic data of 141,614 individuals from six major populations resulted in the most comprehensive map of Kv11.1 variability and its ethnogeographic distribution. Importantly, by cross-referencing genetic variation with ECG and drug exposure data from patients undergoing DHA-PQ therapy in Mali, we identified two common and three rare variants associated with clinically significant drug-induced QT prolongations. These results indicate that genetic variation in *KCNH2* might serve as useful biomarkers for the personalized prediction of cardiac safety of piperaquine and possibly other drugs with Kv11.1-interacting pharmacophores.

## Data Availability

Aggregated population-scale variability data of *KCNH2* can be accessed via the publicly available gnomAD repository (https://gnomad.broadinstitute.org/).

## SUPPLEMENTAL MATERIAL

Figure S1 aac.01390-23-s0001.eps Flow chart depicting the analyzed patient sub-cohorts from WANECAM.

Figure S2 aac.01390-23-s0002.eps Variation in QTc from day 0 to day 2 (ΔQTc) when also individuals without available exposure data are considered.

Table S1 aac.01390-23-s0003.docx Frequencies of KCNH2 variants in the two different study sites.

Table S2 aac.01390-23-s0004.docx Baseline demographic and clinical features of the analyzed trial cohort.

Table S3 aac.01390-23-s0005.xlsx List of variants at the KCNH2 locus that are in linkage with rs1805121 and rs41314375.

ASM does not own the copyrights to Supplemental Material that may be linked to, or accessed through, an article. The authors have granted ASM a non-exclusive, world-wide license to publish the Supplemental Material files. Please contact the corresponding author directly for reuse.
