# Supplementary material for: Population-specific variations in KCNH2 predispose patients to delayed ventricular repolarization upon dihydroartemisinin-piperaquine therapy
Source: Antimicrob Agents Chemother. 2024 Mar 28;68(5):e01390-23. doi: 10.1128/aac.01390-23 (PMC11064487; doi:10.1128/aac.01390-23)
Supplement: Table S1 — Frequencies of KCNH2 variants in the two different study sites. [file aac.01390-23-s0003.docx]

**Supplementary Table 1. Frequencies of *KCNH2* variants in the two different study sites.** Freq = variant frequency; HGVS = Human Genome Variation Society.

| **Rs ID** | **HGVS Consequence**  **NM_000238.4** | **Protein change** | **Freq Kollé (%)** | **Freq Bougoula (%)** |
| --- | --- | --- | --- | --- |
| rs1805121 | c.1692A>G | p.Leu564= | 80.41 | 85.16 |
| rs41314375 | c.1809C>T | p.Gly603= | 4.12 | 5.21 |
| rs1137617 | c.1956T>C | p.Tyr652= | 95.79 | 95.64 |
| rs1335240034 | c.1653C>G | p.Phe551Leu | 0.53 | - |
| rs199473517 | c.1689G>T | p.Trp563Cys | 0.53 | - |
| rs1460801598 | c.1701C>G | p.Ile567Met | 0.53 | - |
| rs121912508 | c.1744C>T | p.Arg582Cys | 0.53 | - |
| rs150275982 | c.1581G>A | p.Ala527= | - | 0.26 |
| rs2116964399 | c.1596G>C | p.Leu532= | - | 0.26 |
| Chr7:150951757 | c. 1637G>A | p.Gly= | 0.53 | - |
| Chr7:150951789 | c. 1605A>T | p.Val535Glu | - | 0.26 |
| Chr7:150951078 | c. 1988A>T | p.Ile663= | - | 0.34 |
| Chr7:150950948 | c. 2118C>A | p.Ser706= | - | 0.34 |
