# Supplementary material for: Population-specific variations in KCNH2 predispose patients to delayed ventricular repolarization upon dihydroartemisinin-piperaquine therapy
Source: Antimicrob Agents Chemother. 2024 Mar 28;68(5):e01390-23. doi: 10.1128/aac.01390-23 (PMC11064487; doi:10.1128/aac.01390-23)
Supplement: Table S2 — Baseline demographic and clinical features of the analyzed trial cohort. [file aac.01390-23-s0004.docx]

**Supplementary Table 2. Baseline demographic and clinical features of the analyzed trial cohort.** IQR = interquartile range; SD = standard deviation.

| Parameters | With drug level | Without drug level | Significance |
| --- | --- | --- | --- |
| **Number of patients** | 69 | 129 |  |
| **Age in years (SD)** | 8 (3.3) | 8 (3.7) | p = 0.128^a^ |
| < 5 | 13 (18.8 %) | 22 (17.6 %) | p = 0.753^b^ |
| ≥ 5 to < 15 | 55 (79.7 %) | 99 (79.2 %) |  |
| ≥ 15 | 1 (1.5 %) | 4 (0.8 %) |  |
| **Female sex** | 35 (50.7 %) | 61 (48.6%.) | p = 0.797^b^ |
| **Body weight in kg (SD)** | 20.42 (8.1) | 22.60 (9.4) | p = 0.119^c^ |
| **Fever present** | 66 (95.7 %) | 121 (96.8 %) | p = 0.907^b^ |
| **Body temperature in °C (SD)** | 38.1 (1.1) | 37.9 (1.2) | p = 0.247^a^ |
| ***Plasmodium falciparum* asexual forms** | 68 (98.7 %) | 120 (96 %) | p = 0.326^b^ |
| Median number of parasites per µL (IQR) | 26290 [12675- 49710] | 10030 [605 – 38140] | p = 0.002^c^ |
| ***Plasmodium ovale* asexual forms** | 0 | 0 | - |
| Median number of parasites per µL (IQR) | - | - | - |
| ***Plasmodium malariae* asexual forms** | 2 (2.9 %) | 11 (8.8 %) | p = 0.393^b^ |
| Median number of parasites per µL (IQR) | 3540 [200 - 6880] | 1060 [80 – 4140] | p = 0.744^c^ |
| **Patients with gametocytes** |  |  |  |
| *Plasmodium falciparum* | 2 (2.9 %) | 0 | - |
| *Plasmodium malariae* | 0 | 0 |  |
| *Plasmodium ovale* | 0 | 0 |  |

^a^ unpaired t-test

^b^ χ^2^ test

^c^ Mann-Whitney U test
